# Systemic Inflammatory Index in Polycythemia Vera and Its Prognostic Implications

**DOI:** 10.3390/jcm13154459

**Published:** 2024-07-30

**Authors:** Ivan Krecak, Danijela Lekovic, Isidora Arsenovic, Andrija Bogdanovic, Hrvoje Holik, Ivan Zekanovic, Martina Moric Peric, Marko Lucijanic

**Affiliations:** 1 Department of Internal Medicine, General Hospital of Sibenik-Knin County, 22000 Sibenik, Croatia; 2 Faculty of Medicine, University of Rijeka, 51000 Rijeka, Croatia; 3 University of Applied Sciences, 22000 Sibenik, Croatia; 4 Clinic of Hematology, University Clinical Center of Serbia, 11000 Belgrade, Serbia; 5 Faculty of Medicine, University of Belgrade, 11000 Belgrade, Serbia; 6 Department of Internal Medicine, Dr. Josip Benčević General Hospital, 35000 Slavonski Brod, Croatia; 7 Department of Internal Medicine, General Hospital Zadar, 23000 Zadar, Croatia; 8 Division of Hematology, University Hospital Dubrava, 10040 Zagreb, Croatia; 9 School of Medicine, University of Zagreb, 10000 Zagreb, Croatia; 10 Department of Scientific Research and Translational Medicine, University Hospital Dubrava, 10040 Zagreb, Croatia

**Keywords:** systemic inflammatory index, myeloproliferative neoplasms, thrombosis, survival, cardiovascular risk

## Abstract

**Background**: This study aimed to evaluate the clinical and prognostic associations of the systemic inflammatory index (SII) in polycythemia vera (PV) patients. SII integrates information on absolute neutrophil (ANC), lymphocyte (ALC), and platelet counts into one index (calculated as ANCxALC/platelet count) and was previously shown to predict thrombotic and mortality risks in the general population. **Methods**: A total of 279 PV patients treated in several hematologic centers in Croatia and Serbia was retrospectively evaluated. **Results**: The median SII for the overall cohort was 1960. Higher SII stratified at the specific cut-off points was significantly associated with shorter time to thrombosis (TTT; *p* = 0.004) driven by arterial thrombotic events, and shorter overall survival (OS; *p* < 0.001). Higher SII was able to refine the European Leukemia Net-defined high-risk patient subgroup for both thrombotic and survival risks, especially in individuals over 60 years of age. SII and all other evaluated CBC components and indices (leukocytes, ANC, ALC, platelets, neutrophil to lymphocyte ratio (NLR), and platelet to lymphocyte ratio (PLR)) demonstrated low-to-modest prognostic properties, whereas SII outperformed other parameters with respect to TTT and OS prognostications. **Discussion**: The presented results complement prior studies evaluating the prognostic performance of different CBC components for thrombotic and survival risk predictions and offer more options to personalize PV treatments.

## 1. Introduction

Polycythemia vera (PV) is a *BCR::ABL1*-negative myeloproliferative neoplasm (MPN) characterized by constitutive *JAK-STAT* signaling due to acquired mutations in the *JAK2* gene, which is present in the majority of patients (>90%), leading to elevated red cell mass, increased blood viscosity, chronic inflammatory state, and high cardiovascular risk [1]. Approximately one-third of PV patients will experience a cardiovascular event during their lifetime, which may lead to impaired overall survival (OS) [2]; however, with optimal management, OS in PV may be near normal [3]. Therapeutically, all PV patients are periodically phlebotomized to reduce red cell mass and receive primary or secondary thromboprophylaxis with aspirin as these two interventions have been shown to reduce the risk of thrombotic events in randomized clinical trials [1,4,5].

Risk stratification in PV is focused mainly on predicting future thrombotic events. The European Leukemia Network (ELN) criteria identify high-risk patients as those older than 60 years of age or with prior thrombosis: these patients are additionally managed with cytoreduction (usually hydroxyurea or interferon alpha) [6]. Despite all these interventions, a substantial residual risk of thrombosis in PV still exists [7] and remains an unmet clinical need. Therefore, the refinement of the ELN risk stratification in PV currently represents an active area of research on MPNs with an aim to delineate PV patients at different risks of future thrombotic events; this approach may potentially offer more personalized therapeutic approaches.

Recently, the superior performance for risk stratification in PV was shown when including molecular mutations in the Mutation-Enhanced International Prognostic System (MIPPS) [8]; however, its use has not been widely adopted, mostly due to its unavailability and cost. For this reason, the scientific community has largely focused on identifying easily available predictors from the complete blood count (CBC), a main tool in the hematologists workshop [9]; the most notable ones are white blood cell count (WBC) [10,11,12,13], absolute neutrophil count (ANC), absolute lymphocyte count (ALC), platelet count, neutrophil-to-lymphocyte ratio (NLR), platelet-to-lymphocyte ratio (PLR), and red blood cell distribution width (RDW) [14,15,16,17,18,19,20]. Even though the role of high WBC in promoting thrombosis in MPNs has long been suspected, randomized data to support its incorporation into risk stratification have been lacking; however, recent prospective observational data from United States of America (USA) community practices (REVEAL) have demonstrated that leukocytosis may be independently (of hematocrit) associated with higher thrombotic risk, suggesting that controlling leukocytosis may be beneficial [21]. On the other hand, increased myeloproliferation in PV and other MPNs (i.e., potentially behind high ANC, high platelet count, and high serum lactate dehydrogenase—LDH) and pronounced immune activation (i.e., potentially behind low ALC and high absolute monocyte count—AMC) may cause different leukocyte subpopulations to be associated with thrombotic risk and OS in opposite directions in various MPN subsets [22,23,24,25] and cause significant discrepancies during the analysis of retrospective datasets where only WBC has been included as the assessed parameter. In fact, MPNs are characterized by abnormal activity of the immune system, and show an increase in the monocyte/macrophage compartment, lower number of altered regulatory and helper T cells, as well as dysfunctional natural killer (NK) cell activity, all of which may underlie MPN development, mediate disease symptoms, and promote disease progression [26,27,28], Besides a low ALC, a high AMC is also an important part of the innate immune system and was shown to correlate with dismal outcomes in PV [29].

Conversely, the NLR and PLR, which both account for a high ANC, high platelet count, and low ALC, by integrating their values into the final calculation have been consistently associated with inferior outcomes in MPNs [15,16,17]. Similarly, the novel triple A model (AAA) focusing on advanced age, high ANC, and low ALC has been developed by the Mayo Clinic, validated on other cohorts, and was shown to excellently discriminate OS in patients with essential thrombocythemia (ET), an associated MPN [30,31]. Notably, the AAA model was also subsequently validated in PV for the prediction of both inferior OS and time to thrombosis (TTT) [32], whereas it can be used in MF patients for OS prognostication but does not surpass the utilities of standard prognostic scores [33]. Thus, the universal MPN risk model based on the combination of these indices does not seem to be possible, but it may provide highly informative insights into the risks of specific MPN entities.

Another issue is that many MPN patients present exclusively with high ANCs, low ALCs, or high platelet counts, whereas others have different combinations of these indices. Additionally, specific CBC indices may be associated with specific event types, i.e., an association of high NLR and high ANC with the risk of venous but not arterial events [14,16].

The systemic inflammatory index (SII) has demonstrated an association with all-cause and cardiovascular mortality in the general population and it could be prognostically useful in PV, as it integrates all of the aforementioned MPN features through different CBC components (ANC, platelet count, and ALC). It is calculated as (ANC × 10^9^/L × platelet count × 10^9^/L)/ALC × 10^9^/L [34,35]. Therefore, the assessment of the SII may potentially help to identify a larger proportion of PV patients presenting with features of higher myeloproliferation and enhanced immune dysregulation, which could be at higher risk of inferior outcomes. Notably, due to its integration of different CBC components, which reflect these typical MPN features, the SII has also been shown to be able to discriminate PV from other causes of polycythemia [36].

The aim of this study was to investigate the SII in PV patients, its clinical correlations, and the potential prognostic impact of the SII on thrombotic risk and survival.

## 2. Materials and Methods

### 2.1. Patient Selection and Study Design

This international study was conducted at several hematological centers in Croatia and Serbia in the period of 2002–2021. Patients with PV whose disease diagnosis was reassessed according to the 2016 World Health Organization criteria [37] were retrospectively included. Clinical and laboratory data at the time of disease diagnosis were collected through a medical chart review. Comorbidities were assessed cumulatively with the Charlson Comorbidity Index (CCI) [38]. Specific cardiovascular risk factors (CVRFs) of interest were arterial hypertension, diabetes mellitus, hyperlipidemia, chronic kidney disease (CKD), and active smoking. Risk stratification was performed with both ELN criteria [6] and the AAA risk model; the latter assigns 4 points for age >70 years, 2 points for age in the range of 50–70 years, and 1 point each for ANC > 8 × 10^9^/L and ALC < 1.7 × 10^9^/L—the corresponding risk categories include low risk (0–1 points), intermediate 1 risk (3 points), intermediate 2 risk (4 points), and high risk (5–6 points) [30].

### 2.2. Statistics

According to Shapiro–Wilk’s test, the data were not symmetrically distributed, thus nonparametric statistical tests were used. Categorical variables were compared using the chi-squared test and continuous variables were compared with the Mann–Whitney U test. Receiver operating curve (ROC) analysis was used to determine the optimal cut-off values for survival analyses. Univariate survival analyses were performed with the Kaplan–Meier method and the log-rank test, whereas the Cox regression was used for multivariate survival analyses. TTT was measured from the time of disease diagnosis until a thrombotic event or last follow-up visit, with death being a censoring event. Arterial thrombotic events considered were acute myocardial infarction, transitory ischemic attack, ischemic stroke, or acute peripheral arterial occlusion; venous thrombotic events of interest were pulmonary embolism and/or deep vein thrombosis. OS was measured from the time of diagnosis until death or the last follow-up visit. The statistically significant *p* value was set at <0.050 for all presented analyses. All statistical computations were performed with MedCalc Statistical Software^®^ (Ostend, Belgium, version 22.021).

## 3. Results

### 3.1. Patient Characteristics and Associations with the SII

A total of 279 PV patients was included; median age was 66 years (range: 20–92), 131 (47%) were females, the median CCI was 3 (range: 0–8), and 240 (86%) had at least one CVRF. According to the ELN risk score, 201 (72%) of patients were high-risk, whereas 27 (9.7%), 141 (50.5%), 43 (15.4%), and 68 (24.4%) of patients belonged to the low, intermediate-1, intermediate-2 and high-risk categories, respectively, according to the AAA risk model.

The median SII for the overall cohort was 1960 (range: 188.2–29,637). Overall patient characteristics stratified at the median SII value are summarized in Table 1. As shown, patients with a higher SII were older (*p* = 0.006), had higher LDH (*p* = 0.019), more often belonged to higher-risk AAA categories (*p* < 0.001), more frequently required cytoreductive treatment (*p* < 0.001), had more thrombotic events during the follow-up (*p* = 0.018), especially arterial events (*p* = 0.003), and experienced more deaths (*p* = 0.005). Expectedly, more patients with a higher SII had higher ANC and platelet counts, and lower ALCs (*p* < 0.050 for all analyses). When specifically analyzing the SII as a continuous variable among patients treated with different treatment modalities, those managed with phlebotomies only had lower median SII when compared to those who required cytoreductions (median SII: 1482 vs. 2376; *p* < 0.001). There were no statistically significant differences with respect to other clinical and laboratory variables.

### 3.2. Assocations of the SII with Thrombotic Risk and Survival

The median follow-up time was 95.1 months (range: 1.8–213) with 121 (43.4%) deaths and 48 (17.3%) thrombotic events (arterial 33 and 15 venous) occurring during this period of time. Death causes included heart failure (n = 45, 37.2%), thrombotic events (n = 18, 14.8%), solid tumors (n = 17, 14%), infections (n = 2, 1.6%), PV progression (n = 2, 1.6%), and other/unknown (n = 37, 30.5%). There were no statistically significant differences in the frequency of thrombotic events during the follow-up between patients who received aspirin (n = 40/232, 17.2%) and those who did not (n = 8/47, 17%; *p* = 0.971).

**Figure 1:**
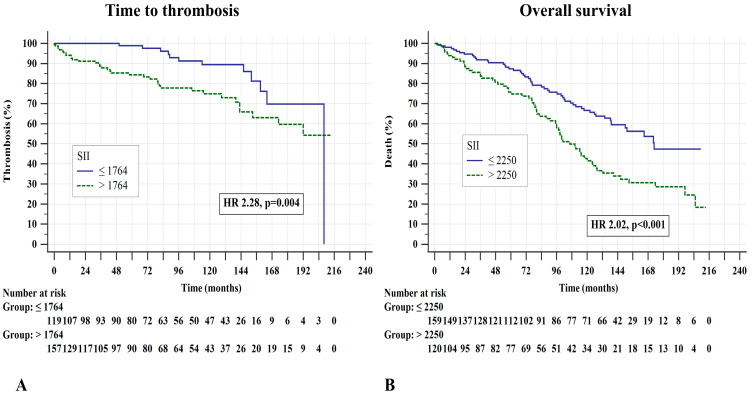
(**A**) Time to thrombosis and (**B**) overall survival stratified according to the systemic inflammatory index (SII). The Kaplan–Meier method and log-rank tests were used.

Using thrombosis and death as classification variables, we set the optimal ROC-defined cut-off values of the SII for the prognostications of composite thrombosis, arterial events, venous events, and death at >1764, >1844, ≤1500, and >2250, respectively. As shown in Figure 1, patients with a higher SII had an inferior TTT (median not reached vs. 208 months, HR 2.28, *p* = 0.004; Figure 1A) and lower OS (median 106.4 vs. 172.7 months, HR 2.02, *p* < 0.001; Figure 1B).

High (>1844) SII was also associated with a shorter time to arterial (HR 2.30, *p* < 0.001) but not venous events (*p* = 0.778). Other variables being associated with an inferior TTT were high (>4) AAA risk-score category (HR 5.9, *p* < 0.001), age >70 years (HR 3.34, *p* < 0.001), presence of CKD (HR 2.07, *p* = 0.045), high (>12.7 × 10^9^/L) WBC (HR 1.96, *p* = 0.028), high (>5.8 × 10^9^/L) ANC (HR 2.1, *p* = 0.025), low (≤2 × 10^9^) ALC (HR 1.88, *p* = 0.028), high (>2.8) NLR (HR 2.17, *p* = 0.011), and high (>221) PLR (HR 2.28, *p* = 0.004), whereas the ELN risk score was of borderline significance (HR 1.68, *p* = 0.080). Notably, none of the CVRFs factors was associated with an inferior TTT or OS; however, higher SII was prognostic of an inferior TTT both in patients with and without CVRFs (*p* < 0.050 for both analyses) and was also prognostic of an inferior OS in patients with CVRFs (*p* < 0.001). Statistically significant interactions existed in high-risk disease between the presence of CVRFs, high SII (*p* = 0.023), and an inferior TTT, and were also of borderline significance in low-risk PV (*p* = 0.055).

Survival was predicted by the ELN (HR 3.22, *p* < 0.001) and the AAA risk scores (overall *p* < 0.001), age > 60 years (HR 3.50, *p* < 0.001), presence of CKD (HR 2.38, *p* < 0.001), higher CCI (*p* < 0.001), high (>12.7 × 10^9^/L) WBC (HR 2.07, *p* = 0.002), high (>10.8 × 10^9^/L) ANC (HR 3.24, *p* < 0.001), high (>578 × 10^9^/L) platelet count (HR 1.44, *p* = 0.044), high (>3.4) NLR (HR 1.89, *p* = 0.004), and high (>580) PLR (HR 3.33, *p* = 0.001).

**Figure 2:**
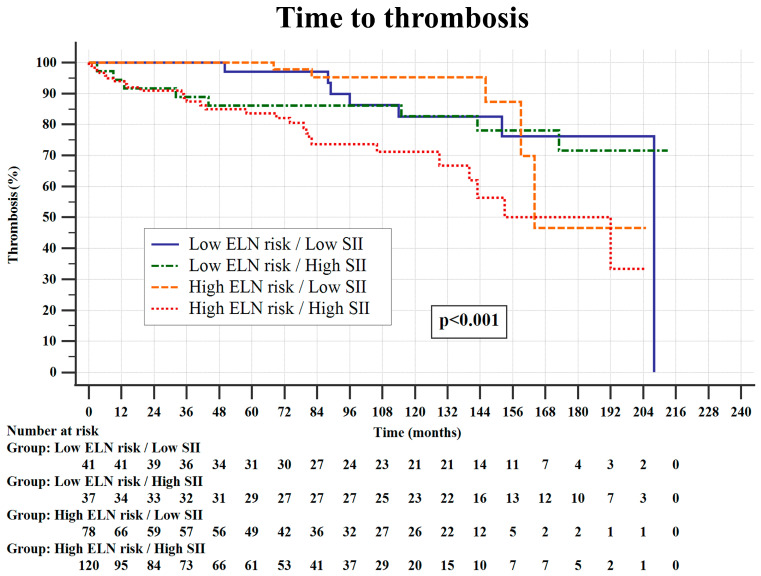
Time to thrombosis stratified according to the European Leukemia Network (ELN) risk score and systemic inflammatory index (SII; cut-off value of 1764). The Kaplan–Meier method and log-rank tests were used.

When patients were stratified according to both ELN risk score and the SII (cut-off 1764), only ELN high-risk patients with high SIs had an inferior TTT when compared to other patient subgroups, which had comparable thrombotic risks over time (*p* < 0.001), as shown in Figure 2.

As the SII does not account for age or prior thrombosis (in contrast to the ELN and the AAA risk scores), we further analyzed its performance in these specific patient subgroups. In patients without prior thrombosis, high SII was able to predict an inferior TTT (HR 2.23, *p* = 0.016), but its prognostic properties were limited in those with prior thrombosis (HR 2.29, *p* = 0.199). High SII was also able to discriminate thrombotic risk in patients older than 60 years of age (HR 3.76, *p* < 0.001) but not in those younger (*p* = 0.943); the results remained the same when age was stratified at 70 years as in the AAA model.

Similarly to thrombotic risk, a higher (>2250) SII was able to discriminate OS among ELN high-risk (HR 2.19, *p* = 0.001) but not in low-risk patients (*p* = 0.361). High SII was also able to delineate OS in patients older than 60 years of age (HR 2.05, *p* < 0.001) but not in those younger (*p* = 0.936). Notably, high SII was able to prognosticate inferior OS both in patients with (HR 2.52, *p* = 0.005) and without prior thrombosis (HR 1.77, *p* = 0.011).

We then performed a series of multivariate Cox regression analyses to assess how the SII compares to the ELN risk stratification and the AAA model with respect to thrombotic and survival predictions when being additionally adjusted for other clinically meaningful variables. As shown in Table 2, higher SII (HR 7.54, *p* = 0.006) outperformed the ELN risk score (*p* = 0.117) with respect to thrombotic risk prognostication, whereas it provided mutually independent prognostic properties (HR 7.80, *p* = 0.005) along with the ELN score (HR 7.59, *p* = 0.005) and the CCI (HR 15.72, *p* < 0.001) with respect to survival prediction.

When being assessed together with the AAA risk score, both high SII (HR 3.98, *p* = 0.046) and the AAA model (HR 8.88, *p* = 0.003) provided mutually independent information with respect to thrombotic risk, whereas the triple A model (HR 15.76, *p* < 0.001) and the CCI (HR 16.77, *p* < 0.001) demonstrated mutually independent prognostic properties and outperformed the SII with respect to survival prediction. When age was treated as a continuous variable, both advanced age (HR 8.54, *p* = 0.003) and high SII (HR 7.61, *p* = 0.005) were independently of each other associated with an inferior TTT when being adjusted for sex, prior thrombosis, CVRFs, and cytoreductive treatment (*p* > 0.050 for all analyses). There were also statistically significant interactions between being older in age, high SII, and thrombotic risk (*p* < 0.001), demonstrating that both advanced age and high SII may mutually contribute to CV disease development in PV patients.

### 3.3. Comparison of the SII with Other Complete Blood Cell Count Indices

Finally, we aimed to evaluate how SII relates to other frequently used blood cell counts and indices, WBC, ANC, ALC, platelet count, NLR, and PLR with respect to thrombotic risk and OS prognostication. Table 3 summarizes the ROC-defined cut-off points, associated risks, and the Harrell’s C indexes for all evaluated indices. As shown, all the aforementioned indices and their ROC-defined cut-off points demonstrated overall low to modest prognostic properties, stratified patients into considerably differently sized prognostic subgroups, and were unable to predict venous events. As mentioned previously, the ROC-defined cut-off values of higher WBC, higher ANC, higher NLR, PLR, and SII were associated with higher thrombotic risk (composite and arterial) and an inferior OS, whereas the lower ROC-defined cut-off value for ALC demonstrated an association with composite thrombotic events and higher platelet count with an inferior OS. When these CBC indices with their corresponding ROC-defined cut-off points were evaluated simultaneously in a multivariate Cox regression model, low (≤2 × 10^9^/L) ALC (*p* = 0.041) and high (>10.8 × 10^9^/L) ANC (*p* = 0.002) remained as the only predictors of an inferior TTT and OS, respectively, possibly due to their overlapping prognostic properties. It should be noted, however, that Harrel’s C indexes for the SII were higher than those of other CBC components for the prognostication of composite (0.648) and arterial thrombotic events (0.672) as well as for OS (0.585).

## 4. Discussion

This is the first study to investigate prognostic implications of SII in PV patients and to demonstrate that its higher values may be associated with an inferior TTT and OS, suggesting that SII may be used for both diagnostic [36] and prognostic purposes. Considering that SII has been associated with cardiovascular disease development in the general population [34,35] and the fact that it accounts for the typical MPN features—increased myeloproliferation (high ANC, platelet count, and higher LDH) and immune dysregulation (low ALC), we found it relevant to evaluate its prognostic implications in PV. In fact, the SII outperformed other CBC indices with respect to thrombosis and survival prognostications, suggesting that the SII may provide a more comprehensive measure of the aforementioned underlying pathophysiological processes in PV. Interestingly, the SII did not correlate with any of the CVRFs nor other patient-related characteristics, aside from the CBC components, suggesting that it may primarily reflect these intrinsic MPN features. With this in line, patients with high SII more often needed cytoreductive treatment, which may potentially suggest a lower degree of myeloproliferation and less aggressive disease in those managed only with phlebotomies. It should be pointed out, however, that all investigated CBC indices demonstrated mostly low to moderate prognostic properties with respect to thrombotic and survival predictions. On the other hand, this study adds to the increasing pool of evidence that leukocytosis, high ANC, low ALC, and high NLR and PLR may be associated with inferior outcomes in MPNs. Actually, the evaluated cut-off values of ANC, NLR, and PLR correspond to those from our prior studies [15,22], whereas they were only slightly different in comparison to their corresponding cut-offs from the USA and Italy [14,16], albeit they correlated in the same direction with respect to thrombotic risk. Again, high platelet count did not show a univariate correlation with higher thrombotic risk, which is in line with previously published reports [39], but did with an inferior OS. This observation suggests that integrating platelet count into SII calculations (for example, in contrast to the NLR) may help “to capture” more PV patients with an increased risk of inferior outcomes and thus provide additional prognostic information. Also, none of the evaluated indices was associated with venous events, suggesting that different pathophysiological processes may be involved in the promotion of specific thrombotic event types in PV [40]. In line with this is an observation that cytoreductive treatment, which affects the CBC count, leukocyte–platelet interactions, and the endothelium, seems to be more potent in arterial than in the venous district [41,42]. Finally, the prognostic role of the SII is yet undisclosed among MF patients since only limited insights have been published to date [43]. It should be noted that lower ALCs and platelet counts are associated with higher thrombotic risk in overt MF patients, which is in contrast to prefibrotic MF where this risk is predicted by a higher ANC, higher ALC, and higher platelet count [22].

Considering the current study, the associations of a higher SII with thrombotic risk persisted in multivariate models when being adjusted for both ELN and the AAA risk scores. On the other hand, higher SII remained independent of the ELN score and comorbidities associated with an inferior OS, whereas the AAA risk model outperformed the SII with respect to survival prediction. The observation that the ELN score could not be associated with higher thrombotic risk in the presented study could be explained by the fact that cytoreductive treatment in this retrospective study was mostly driven by this particular score potentially diminishing its prognostic properties. On the other hand, the AAA model outperformed the SII with respect to OS prediction—this observation may be due to the larger prognostic weight given to advanced age in the AAA model and the fact that both the SII and AAA prognostically overlap, as both of them incorporate ANC and ALC. Nevertheless, the SII was able to further refine the high-risk ELN patient subgroup with respect to thrombotic and survival risks; accordingly, a high SII could potentially help to identify a subset of high-risk PV patients who may require more intensive cytoreduction, switch to other regimens, such as interferons or ruxolitinib [44,45,46], twice-daily aspirin [47], or a more rigorous phlebotomy schedule. Furthermore, the SII performed particularly well in two other important patient subgroups, patients without prior thrombosis and those older than 60 years of age, suggesting that these particular patient groups may also benefit from an SII assessment at the time of disease diagnosis. These patients are sometimes witheld from cytoreduction as the only indication for treatment is advanced age (i.e., 62 years of age in an otherwise healthy individual). Therefore, the calculation of the SII may potentially help to identify a proportion of PV patients for which this treatment strategy may be feasible. An observation that the SII did not perform well in PV patients with prior thrombosis history could be explained by the fact that these patients receive cytoreductive treatment and different potent cardioprotective medications for CVRF control and secondary prevention (i.e., angiotensin-converting enzyme inhibitors (ACE-is), statins, sodium–glucose cotransporter-2 (SGLT2) inhibitors, anticoagulants, etc.), which could modulate their thrombotic risk over time. Also, the long follow-up time over which PV patients were recruited, evolving CVRF diagnostic criteria and changing treatment targets for different metabolic deflections (i.e., for the control of blood lipids) and other intrinsic biases specific to retrospective datasets, may have caused none of the CVRFs to be associated with inferior outcomes in our study [48,49,50]. Finally, high SII was associated with arterial but not venous events—this observation is most probably due to the small number of venous events, which could have affected the statistical power, as well as due to high frequency of CVRFs, which are more frequently associated with arterial thrombotic events (i.e., 86% of patients included in this study had at least one CVRF; 75% had arterial hypertension; and 25% were active smokers). On the other hand, high SII was prognostic of an inferior TTT both in patients with and without CVRFs, with statistically significant interactions between having CVRF, high SII, and higher thrombotic risk, both in low- and high-risk disease, suggesting that the SII may help to further refine the prognosis in different PV patient populations. Nevertheless, additional studies are needed to validate these findings.

The main limitations of this study include its retrospective design, relatively limited number of patients, especially in different patient subgroups, and the absence of cytogenetic and molecular data. Also, due to the low number of disease progressions during the follow-up, we could not evaluate the potential role of the SII in predicting disease transformation. Additionally, validation on larger datasets, among demographically different populations, and in other MPN subtypes, is needed. Finally, disease risk could shift over time (i.e., due to increasing age or after a thrombotic event) and longitudinal SII assessments may be needed to test whether the SII could be potentially used as a dynamic parameter to improve the prognostication of PV patients and to guide treatments. Further studies are needed to focus on these remaining issues.

## 5. Conclusions

In patients with PV, a higher SII may be associated with an inferior TTT and OS; the association of a high SII with thrombotic risk outperformed the ELN score and was independently associated with an inferior OS, whereas higher SII, the AAA risk model, and the CCI independently of each other predicted thrombotic events. Conversely, the AAA risk score outperformed the high SII with respect to OS prediction. Higher SII was also able to further refine the ELN high-risk patient subgroup for both thrombotic and survival risks, especially in individuals over 60 years of age. Finally, even though all evaluated CBC indices demonstrated low to modest prognostic properties, the SII outperformed other evaluated parameters (WBC, ANC, ALC, platelet count, NLR, and PLR) with respect to TTT and OS predictions. Therefore, integration of these three CBC components (ANC, ALC, and platelets) into a comprehensive composite measure, such as the SII, may help to further improve the risk stratification of PV patients. The presented results compliment prior studies evaluating the prognostic performance of different CBC components for thrombotic and survival risk predictions and offer more options to personalize PV treatments.

## Figures and Tables

**Table 1:** Overall patient characteristics stratified according to the median systemic inflammatory index (SII) value. The chi-squared and Mann–Whitney U tests were used.

Variable	Overall (n = 279)	Low SII (n = 142, 50.9%)	High SII (n = 137, 49.1%)	*p*
Age (years)	66 (20–92)	63.5 (20–92)	68 (31–85)	0.006 *
Sex (female)	131 (47%)	67 (47.2%)	64 (46.7%)	0.937
Prior thrombosis	66 (23.7%)	32 (22.5%)	34 (24.8%)	0.654
Splenomegaly	41 (14.7%)	19 (13.4%)	22 (16.1%)	0.528
Constitutional symptoms (n = 175)	49 (28%)	22 (25%)	27 (31%)	0.375
ELN high risk	201 (72%)	96 (67.6%)	105 (76.6%)	0.093
AAA				<0.001 *
Low	27 (9.7%)	16 (59.3%)	11 (40.7%)	
Intermediate-1	141 (50.5%)	92 (65.2%)	49 (34.8%)	
Intermediate-2	43 (15.4%)	15 (34.9%)	28 (65.1%)	
High	68 (24.4%)	19 (27.9%)	49 (72.1%)	
CCI	3 (0–8)	3 (0–8)	3 (0–7)	0.762
Arterial hypertension	208 (74.6%)	109 (76.8%)	99 (72.3%)	0.389
Hyperlipidemia	44 (15.8%)	26 (18.3%)	18 (13.1%)	0.236
Diabetes mellitus	42 (15.1%)	26 (18.3%)	16(11.7%)	0.122
Active smoking	70 (25.1%)	30 (21.1%)	40 (29.2%)	0.120
Chronic kidney disease (n = 261)	57 (21.8%)	26 (19.4%)	31 (24.4%)	0.328
Cytoreductive treatment	196 (70.2%)	83 (58.5%)	113 (82.5%)	<0.001 *
Aspirin	232 (83.2%)	112 (78.9%)	120 (87.6%)	0.052
WBC, ×10^9^/L (median, range)	11.5 (4.2–52.8)	10 (4.2–52.8)	13.5 (5.3–45.8%)	<0.001 *
ANC, ×10^9^/L (median, range)	7.78 (0.53–33.89)	6.42 (0.53–29.56)	10.1 (0.74–33.89)	<0.001 *
ALC, ×10^9^/L (median, range)	2.1 (0.15–5.42)	2.3 (0.17–5.47)	1.9 (0.15–5.2)	<0.001 *
Erythrocytes, ×10^12^/L (median, range)	6.4 (4.37–9.5)	6.4 (4.37–8.8)	6.4 (4.5–9.5%)	0.289
Hematocrit, % (median, range)	53 (43–72)	52 (43–67)	52 (44–72)	0.747
Hemoglobin, g/L (median, range)	173 (136–230)	171 (141–223)	175 (136–230)	0.967
Platelets, ×10^9^/L (median, range)	557 (134–2644)	409 (144–1032)	688 (134–2644)	<0.001 *
LDH, IU/L (median, range)	465 (238–1307)	438 (238–1307)	490 (246–926)	0.019 *
Thrombosis during follow-up	48 (17.3%)	17 (12%)	31 (22.6%)	0.018 *
- arterial	33 (11.8%)	9 (6.3%)	24 (17.5%)	0.003 *
- venous	15 (5.4%)	8 (5.6%)	7 (5.1%)	0.846
Death during follow-up	121 (43.5%)	50 (35.2%)	71 (51.8%)	0.005 *

* Statistically significant at <0.050. SII = Systemic Inflammatory Index, ELN = European Leukemia Network, AAA = Age, Absolute Neutrophil Count—ANC, Absolute Lymphocyte Count—ALC, WBC = White Blood Cell Count, LDH = Lactate Dehydrogenase, IU/L = International Units per Liter, CCI = Charlson Comorbidity Index.

**Table 2:** Multivariate Cox regression analysis for predictions of thrombotic risk and survival.

**Time to Thrombosis**							
*Variable*	*HR*	*95% CI*	*p*	*Variable*	*HR*	*95% CI*	*p*
SII > 1764	7.54	1.31–5.16	0.006 *	SII > 1764	3.98	1.01–4.14	0.046 *
ELN high-risk	2.42	0.87–3.23	0.117	AAA	8.88	1.12–1.73	0.003 *
CVRFs	1.03	0.60–4.85	0.376	CVRFs	1.20	0.66–3.63	0.272
Sex	0.10	0.62–1.93	0.751	Sex	0.1	0.63–5.0	0.779
Cytoreduction	0.45	0.38–1.59	0.498	Cytoreduction	0.58	0.37–1.53	0.445
				Prior thrombosis	0.1	0.51–2.01	0.954
**Overall Survival**							
*Variable*	*HR*	*95% CI*	*p*	*Variable*	*HR*	*95% CI*	*p*
SII > 2250	7.80	1.93–2.73	0.005 *	SII > 2250	1.93	0.87–2.12	0.163
ELN high risk	7.59	1.30–4.74	0.005 *	AAA	15.76	1.19–1.67	<0.001 *
CCI	15.72	1.33–2.36	<0.001 *	CCI	16.77	1.14–1.47	<0.001 *
Sex	2.61	0.47–1.07	0.105	Sex	1.85	0.49–1.13	0.173
Cytoreduction	0.22	0.48–1.55	0.637	Cytoreduction	0.04	0.59–1.89	0.835

* Statistically significant *p* values are set at <0.050, HR = hazard ratio, CI = confidence interval, ELN = European Leukemia Network, CVRFs = presence of cardiovascular risk factors (arterial hypertension, diabetes mellitus, hyperlipidemia, chronic kidney disease, and active smoking), AAA = age, absolute neutrophil count, CCI = Charlson Comorbidity Index.

**Table 3:** Overview of specific cut-offs for complete blood count indices and their prognostic properties. The receiver operating curve (ROC) analysis, the Kaplan–Meier method, and log-rank tests were used.

Variable	Time to Composite Thrombosis	Time to Arterial Thrombosis	Time to Venous Thrombosis	Overall Survival
WBC (×10^9^/L)				
ROC defined cut-off	>12.7	>11.9	≤9.2	>12.7
Proportion of patients	107 (38.4%)	133 (47.7%)	71 (25.4%)	107 (38.4%)
Associated risk	HR 1.96, *p* = 0.028 *	HR 2.31, *p* = 0.020 *	HR 1.18, *p* = 0.763	HR 2.07, *p* = 0.002 *
Harrell’s C index	0.582	0.614	0.500	0.569
ANC (×10^9^/L)				
ROC defined cut-off	>5.8	>9.4	>5.8	>10.8
Proportion of patients	208 (74.6%)	103 (36.9%)	208 (74.6%)	68 (24.4%)
Associated risk	HR 2.1, *p* = 0.025 *	HR 3.12, *p* = 0.003 *	HR 2.87, *p* = 0.067	HR 3.24, *p* < 0.001 *
Harrell’s C index	0.588	0.612	0.616	0.579
ALC (×10^9^/L)				
ROC defined cut-off	≤2	≤2.1	≤1.8	>2.3
Proportion of patients	133 (47.7%)	137 (49.1%)	96 (34.4%)	104 (37.3%)
Associated risk	HR 1.88, *p* = 0.028 *	HR 1.83, *p* = 0.092	HR 1.50, *p* = 0.463	HR 1.1, *p* = 0.604
Harrell’s C index	0.570	0.567	0.603	0.522
PLT (×10^9^/L)				
ROC defined cut-off	>415	>415	≤693	>578
Proportion of patients	196 (70.3%)	196 (70.3%)	196 (70.3%)	129 (46.2%)
Associated risk	HR 1.41, *p* = 0.287	HR 1.80, *p* = 0.139	HR 1.82, *p* = 0.273	HR 1.44, *p* = 0.044 *
Harrell’s C index	0.539	0.511	0.527	0.548
NLR				
ROC defined cut-off	>2.8	>3.8	>2.4	>3.4
Proportion of patients	191 (68.5%)	138 (48%)	218 (78.1%)	156 (55.9%)
Associated risk	HR 2.17, *p* = 0.011 *	HR 3.73, *p* < 0.001 *	HR 2.82, *p* = 0.075	HR 1.89, *p* = 0.004 *
Harrell’s C index	0.608	0.637	0.607	0.568
PLR				
ROC defined cut-off	>221	>256	≤243	>580
Proportion of patients	170 (60.9%)	143 (51.3%)	125 (44.8%)	21 (7.5%)
Associated risk	HR 2.02, *p* = 0.016 *	HR 2.37, *p* = 0.016 *	HR 1.23, *p* = 0.686	HR 3.33, *p* = 0.001 *
Harrell’s C index	0.613	0.638	0.468	0.531
SII				
ROC defined cut-off	>1764	>1844	≤1500	>2250
Proportion of patients	158 (56.6%)	152 (54.5%)	93 (33.3%)	120 (43%)
Associated risk	HR 2.28, *p* = 0.004 *	HR 2.30, *p* < 0.001 *	HR 1.16, *p* = 0.778	HR 2.02, *p* = 0.002 *
Harrell’s C index	0.648	0.672	0.462	0.585

* Statistically significant *p* values are set at <0.050, HR = hazard ratio, WBC = white blood cell count, ROC = receiver operating curve analysis, ANC = absolute neutrophil count, ALC = absolute lymphocyte count, PLTs = platelets, NLR = neutrophil-to-lymphocyte ratio, PLR = platelet-to-lymphocyte ratio, SII = systemic inflammatory index.

## Data Availability

Data are available from the corresponding authors upon reasonable request.
